# Experimental Investigation of Aerosol and CO_2_ Dispersion for Evaluation of COVID-19 Infection Risk in a Concert Hall

**DOI:** 10.3390/ijerph18063037

**Published:** 2021-03-16

**Authors:** Wolfgang Schade, Vladislav Reimer, Martin Seipenbusch, Ulrike Willer

**Affiliations:** 1 Fraunhofer Heinrich Hertz Institute, 38640 Goslar, Germany; vladislav.reimer@hhi.fraunhofer.de; 2 IEPT, Clausthal University of Technology, 38678 Clausthal, Germany; ulrike.willer@tu-clausthal.de; 3 ParteQ GmbH, 76316 Malsch, Germany; seipenbusch@parteq.net

**Keywords:** COVID-19, infection risk, SARS CoV-2 virus, airborne transmission, aerosols, CO_2_ dispersion, concert hall environment

## Abstract

The dispersion of small aerosols in a concert hall is experimentally studied for estimating the risk of infection with SARS-CoV-2 during a concert. A mannequin was modified to emit an air stream containing aerosols and CO_2_. The aerosols have a size distribution with a peak diameter (δ) close to 0.3 µm and a horizontal initial particle velocity ($v_{p,x}$) of 2.4 m/s. The CO_2_-concentration (c) emitted simultaneously is 7500 ppm. It is investigated, if the spatial dissipation of aerosols and CO_2_ can be correlated. This would allow the use of technically easier CO_2_ measurements to monitor compliance with aerosol concentration limits. Both aerosol and CO_2_ concentrations are mapped by different sensors placed around the mannequin. As a result, no significant enrichment of aerosols and CO_2_ was obtained outside a radius of 1.5 m when the fresh air ventilation in the concert hall has a steady vertical flow with a velocity of $v_{g,z}=0.05\;m/s$ and the installed ventilation system was operating at an air change rate per hour (ACH) of 3, corresponding to an air exchange rate of 51,000 m^3^/h. A Pearson correlation coefficient of 0.77 was obtained for CO_2_ and aerosol concentrations measured simultaneously at different positions within the concert hall.

## 1. Introduction

Very recently, the role of aerosols in the transmission of SARS-CoV-2 has been pointed out by several papers [1,2,3,4,5,6]. The spatial distribution of aerosols in an indoor environment is mostly derived from numerical simulation [7,8,9]. However, for public spaces such as concert halls, theaters, or event facilities such data are almost unknown and to date no experimental investigation of aerosol dispersion is reported for such spaces in literature. However, the availability of such data is important in understanding the COVID-19 risk assessment. Therefore, in the present paper experiments have been performed to get more detailed understanding of the spatial distribution of aerosols in a concert hall.

Humans emit respiratory droplets ranging from 0.1 to several tens of micrometers in diameter depending on respiratory activities such as breathing, speaking, or sneezing [10,11,12,13]. Heavier particles are strongly influenced by gravity, and fall down within a few meters, thereby contributing to virus transmission between individuals over relatively short distances. At this point, it needs to be emphasized that physical parameters such as relative humidity, the ambient air temperature, and the intensity of fluid turbulence influence the range and time particles are suspended in air [14,15,16,17,18,19,20,21,22]. Lighter particles (aerosols) in turn can stay in the air for quite a long time and could transfer the virus over larger distances [23,24]. Furthermore, they are reported to reduce in size by evaporation, depending on the content of salts and organic material within the liquid phase. Thus, the ultimate limit in size is the diameter of the virus itself (approx. 0.14 µm for SARS-CoV-2) [13,25,26,27]. Face masks serve to reduce the number of particles, especially heavier ones [17,28,29]. Considering this, more attention should be paid to small particle sizes (diameter < 1 µm) when indoor viral aerosol dispersion is discussed. In addition, CO_2_ measurement is considered as a biomarker for monitoring infectious aerosol occurrence [30,31,32,33]: the lower the indoor CO_2_ concentration, the lower the (potential infectious) aerosol concentration. Therefore, experimental investigation of spatially resolved aerosol and CO_2_ distribution in large indoor environments such as concert halls and theaters with respect to ventilation is highlighted in this paper. Accurate knowledge on dispersion of viral aerosols by airborne routes will be of great value for developing risk assessment when considering re-opening concert halls and theaters after pandemic lock down.

## 2. Materials and Methods

**Figure 1:**
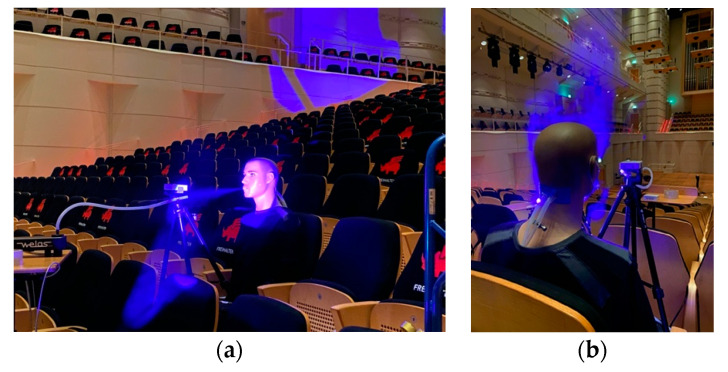
Experimental set-up of the dummy visualizing emitted aerosols by blue light illumination. (**a**) Dummy without a mask. (**b**) Dummy wearing a surgical mask.

**Figure 2:**
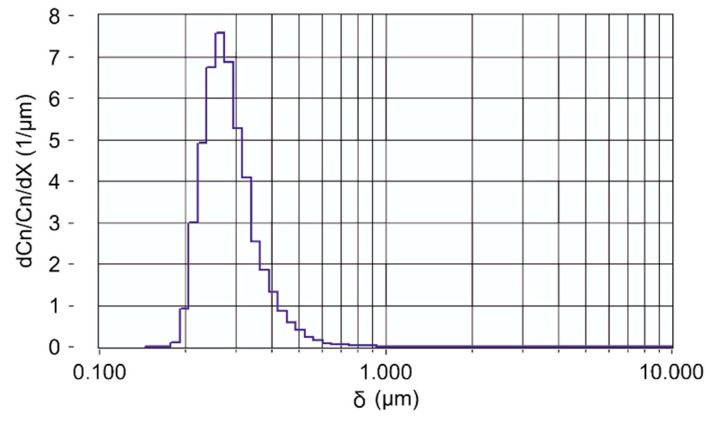
Particle size distribution of emitted DEHS aerosol. δ is the diameter of the particles.

To simulate human emission of aerosols during breathing, a mannequin has been modified as a dummy by introducing tubes to mouth and nose that allow exhalation of aerosols and CO_2_ with a horizontal velocity of $v_{p,x}=2.4\;m/s$ measured 10 cm in front of the mouth. Contrary to a real person, the dummy is emitting aerosols and CO_2_ continuously. The aerosol concentration is adjusted to be about 35,000 p/cm^3^. This is much more than the aerosol concentration exhaled by an individual which varies in the range between 0.1 to 100 p/cm^3^ [34]. A common problem in measuring the dispersion of exhaled aerosols is that the concentration is below the ambient aerosol concentration and decreases rapidly due to dilution and therefore changes are hardly measurable. To circumvent this, a much higher initial concentration is chosen in this study. The emitted CO_2_ concentration is about 7500 ppm. Experiments have been performed with and without a surgical mask (refer to Figure 1). Particles with a well-defined size distribution are generated by an aerosol generator (AGF 2.0ip, Palas GmbH, Karlsruhe, Germany). A typical size distribution of the di-ethylhexyl-sebacate aerosol (DEHS) used during the experiments is shown in Figure 2. The maximum of the size distribution is centered around 0.3 µm. The number of particles emitted by the dummy is monitored as a reference 10 cm in front of the mouth (Palas Promo 2000 and Welas 2300 Sensor) and in parallel a handheld particle counter (Fidas Frog, Palas, with a measuring range 0 to 20,000 p/cm³) is used to monitor the number of emitted aerosol particles at different positions. In addition, also handheld particle counters (PCE-ADQ 20, with a resolution of 1 μg/m³ and a measuring range 0 to 250 μg/m³; PCE-RCM 12, with a resolution of 1 μg/m³ and a measuring range 0 to 2000 μg/m³) are used to monitor time-resolved aerosol concentrations for particles of diameter < 2.5 µm. At the same time the CO_2_ concentration is determined with a battery-powered non-dispersive infrared (NDIR) CO_2_ sensor (LP8, SenseAir AB, Delsbo, Sweden, with a resolution of ±50 ppm and a measuring range 0 to 10,000 ppm) at those positions. The measurements reported in this investigation are performed at Konzerthaus Dortmund, Germany. The volume of the concert hall is about V = 17,000 m^3^, fitting 1650 people. The ventilation system provides a vertical air flow with fresh air entering beneath each of the seats and exiting through the ceiling. The most dominant flow direction is therefore vertically oriented, and the flow velocity is measured to be $v_{g,z}=0.05\;m/s$ while in horizontal direction only a local and temporarily unsteady flow could be measured with a maximum flow velocity of $v_{H}=0.01\;m/s$. The air change rate per hour at 100% power of the installed ventilation system is ACH = 3.

## 3. Results

The aerosol size distribution given in Figure 2 shows a maximum at a diameter (δ)  close to 0.3 µm. Thus, the aerosol distribution emitted by the dummy covers the lower range of particles in human breath as mentioned above. This range is chosen because during a classical concert the audience is sitting quietly without speaking and sneezing. Pöhlker et al. summarized particle size distributions (PSD) measured by numerous authors and fit them separately for different respiratory activities [34]. The dominant mode for breathing peaks at values between diameters of 0.15 µm to 0.53 µm with an average of δ = 0.31 µm [34]. The initial particle velocity of $v_{p,x}=2.4\;m/s$ from the dummy measured at d = 10 cm in front of the face is reduced to $v_{p,x}=0.04\;m/s$ at d = 150 cm by interaction of the aerosols with the air and corresponds at that point to the vertical flow speed of the fresh air ventilation.

To estimate conditions when small diameter aerosols will be taken upwards by the vertical air flow ventilation, a simplistic model has been used to calculate trajectories and to compare those with the measurements. We assume that we can treat the aerosols as single spherical particles with constant diameter δ. We chose a coordinate system oriented such that the x-axis is horizontal in the direction of respiratory flow and the z-axis is pointing downwards. Equations (1) and (2) summarize the forces acting on the particle split into these two components [15,35].

(1) $$m\frac{dv_{p,x}}{dt}=C_{D}\pi \delta^{2}\;\rho_{g}\frac{\left|v_{p}\right|}{8}v_{p,x}$$

(2) $$m\frac{dv_{p,z}}{dt}=g\frac{1}{6}\pi \delta^{3}\;\rho_{g}\left(\frac{\rho_{p}}{\rho_{g}}-1\right)+C_{D}\pi \delta^{2}\;\rho_{g}\frac{\left|v_{p}\right|}{8}v_{p,z}$$

In the x-direction, only the drag force is present, whereas for the z-component the first term is comprised of the gravitational force and the buoyancy force. Here, m  is the mass of the aerosol, $\rho_{g}\;$ the density of the surrounding gas, $\rho_{p}$ the density of the particle or aerosol and $v_{p}\;$ is the particle velocity relative to the surrounding gas; x and z denote the respective components of this vector and $\left|v_{p}\right|=\sqrt{v_{p,x}^{2}+v_{p,z}^{2}}$ its absolute value. The subscripts p and  g denote the properties of the particle/aerosol and the surrounding gas, respectively. $C_{D}$ is the drag coefficient which depends strongly on the Reynolds number. For certain ranges of the Reynolds number, there are piecewise descriptions of the drag coefficient available [36]. The definition for the Reynolds number is given in Equation (3).

(3) $$Re=\frac{\left|v_{p}\right|\delta }{\nu }$$

**Figure 3:**
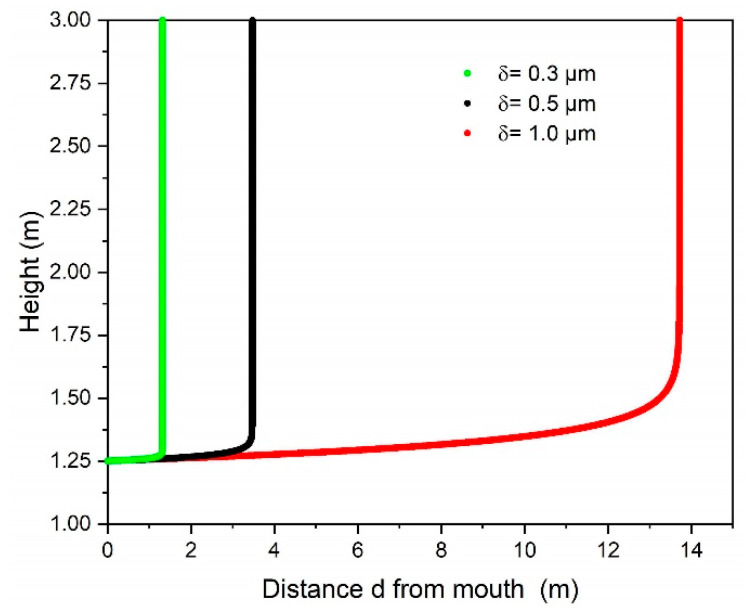
Trajectories of exhaled aerosol particles with different diameter before capturing by a vertical fresh air flow. The initial horizontal exhale velocity of the particles is $v_{p,x}=2.4\;m/s$ (10 cm from the mouth) and the vertical air flow velocity is $v_{g,z}=\;0.05\;m/s$ The material parameters used are: $\rho_{g}=1.189\;kg/m^{3}$, $\nu =15.32\cdot 10^{-6}\;m^{2}/s$ for air and $\rho_{p}=914\;kg/m^{3}$ for DEHS or $\rho_{p}=997\;kg/m^{3}$ for water, respectively. Calculations for DEHS and water aerosols give similar results.

Here, ν is the kinematic viscosity of air. Since both the diameter δ and the relative velocity of the aerosol are small, the value for Re is well below 1 throughout this study. The maximum value reached for a water droplet with 1 µm diameter and an initial relative velocity $\left|v_{p}\right|=2.4\;m/s$ in air (with kinematic viscosity $\nu =\;15.32\;\times 10^{-6}$) is calculated to be Re=0.16. Reynold numbers smaller than 1 are considered as the Stokes limit for which the drag coefficient can be approximated by $C_{D}=\frac{24}{Re}$ [15,36,37]. This simplistic model accounts for a single aerosol droplet within an exterior gas flow, which is appropriate for the vertical motion where there is a widespread vertical airflow from below each seat to the ceiling. However, for the horizontal movement the static surrounding air, the spread of the spatially confined exhaled air flow, its velocity and the initial velocity of the aerosol would be needed for an accurate description. The exhaled air flow prevents the abrupt retardation of the aerosol within the stagnant air. Our simple model is intended for a rough plausibility analysis of the measured values in the present investigation only and neglects the exhaled air flow and thus would overestimate the deceleration; therefore, the influence of the drag force is reduced by an empirically found factor. To determine it, the velocity of the aerosols was measured at different distances and the factor was adapted such that in the model the end velocity was reached at the same distance. For the same settings, the vertical movement could be derived, and trajectories of the particles calculated as depicted in Figure 3. The mouth of the sitting person exhaling is assumed to be at 1.25 m height and the calculation was stopped after reaching a height of 3 m assuming that air from this height is not inhaled by any spectator but is taken to the exhaust at the ceiling. The model does not take any alterations of the drag coefficient due to turbulence into account. The main effect of increased turbulence is that the critical Reynolds number at which the drag coefficient shows an abrupt decrease shifts to lower values [38,39]. However, since in this study the Reynolds number is below 1 and the flow is regarded as creeping flow, it is reasonable to neglect the influence of turbulence. Another simplification concerns the assumption of a fixed diameter—real respiratory aerosols will change their size due to evaporation and depending on the humidity of the surrounding and their composition. However, we concentrate on small particles which can either be produced during normal breathing or which could be originally bigger aerosols that already shrunk and reached their terminal size. There are some investigations about the change of the drag coefficient for evaporating aerosols. For low Reynolds numbers evaporating aerosols show a reduced drag coefficient [40,41,42], thus the model might overestimate the drag.

The differential equations are solved numerically using the software tool mathematica (Wolfram Research Inc., Champaign, IL, USA) for fixed aerosol sizes. The results for 3 different aerosol diameters are summarized in Figure 3. It shows that aerosols with a diameter of δ = 0.3 µm and initial horizontal exhalation velocity of $v_{p,x}=2.4\;m/s$ will be discharged by the vertical ventilation air flow ($v_{g,z}=\;0.05\;m/s$) after a travelling distance of about 1 m and lifted to a height of 3 m. In addition, the diffusion coefficient of CO_2_ molecules in air has been calculated for room temperature and atmospheric pressure as $k_{diff}=1.62\times 10^{-5}\;m^{2}/s$ indicating that the movement of CO_2_ molecules in air can be neglected compared to the velocity of the vertical air flow of the ventilation. This suggests that also the CO_2_ molecules will be captured by the ventilation air flow and directed towards the ceiling.

**Figure 4:**
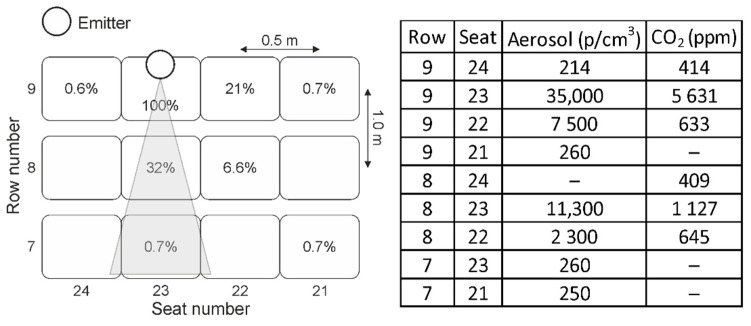
Schematic of seat positions for the dummy and measuring devices. The dummy is not wearing a face mask and emitting aerosols with a concentration of 35,000 p/cm^3^ and CO_2_ of 5631 ppm. A horizontal temporarily fluctuating air flow with $v_{H}=0.01\;m/s$ was present which is directed from the position of the dummy towards row 7/seat 21. The measured CO_2_ and aerosol concentrations are summarized in the table.

In Figure 4, an example for locations and results for the CO_2_ and aerosol measurements in the concert hall is shown when the dummy is not wearing a face mask (refer to Figure 1a). The dummy is located in row 9 at seat number 23, constantly emitting 35,000 p/cm^3^. At a distance of 0.5 m the number density of particles is diluted down to 11,300 p/cm^3^ (row 8/seat 23) and at a distance of 1.5 m it is measured to be 260 p/cm^3^ (row 7/seat 23) which is 0.7 % of the particle number density emitted from the dummy. The seat located directly on the right of the dummy (row 9/seat 24) shows an aerosol concentration of 214 p/cm^3^ while at the seat to the left (row 9/seat 22) a concentration of 7500 p/cm^3^ was obtained and at row 8 seat 22 the concentration was 2300 p/cm^3^, respectively. This is due to the effect that on these positions besides the constant vertical air flow also an unsteady and temporarily fluctuating horizontal air flow with $v_{H}=0.01\;m/s$ was measured, indicating that even a slight change in air flow can result in a significant temporarily dependent change of aerosol dispersion in the neighborhood of an emitter.

However, even if such additional temporarily fluctuating air flow with a horizontal component ($v_{H}=0.01\;m/s$) is present the initial aerosol concentration emitted by the dummy is diluted down to 0.7 % after 1.5 m distance (250 p/cm^3^, row 7 seat 21). The data shown in Figure 4 are averaged 5 min. Parallel to the aerosol measurements the dispersion of CO_2_ was monitored at the same seat positions. A similar pattern is obtained, refer to Figure 4.

**Figure 5:**
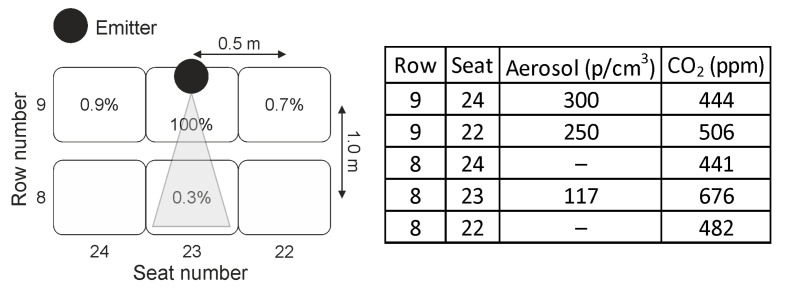
Schematic of seat positions for the dummy and measuring devices. The dummy is wearing a surgical face mask and emits aerosol with a concentration of 35,000 p/cm^3^ and CO_2_ of 5600 ppm. A horizontal temporarily fluctuating air flow with $v_{H}=0.01\;m/s$ was measured which is directed from the position of the dummy towards row 8/seat 22. The measured CO_2_ and aerosol concentrations are summarized in the table.

It has also been investigated which impact on the aerosol and CO_2_ dispersion can be measured when the dummy is wearing a surgical face mask. This aspect is especially interesting keeping the travelling distance of particles emitted at a velocity of $v_{p,x}=2.4\;m/s$ as presented in Figure 3 in mind. Small particles with diameter around δ = 0.3 µm are carried immediately by the vertical air flow as discussed above. Larger particles with a diameter of δ > 1 µm may reach up to a distance of d = 10 m at a nearly constant height (refer to Figure 3 and also [43]). Particle size reduction by evaporation is neglected in this assumption. In the case of a surgical mask worn by the dummy, the exhaled air flow is blocked by the mask and therefore the flow of the aerosols has no major horizontal velocity component and thus is directly dispersed towards the ceiling of the concert hall by the vertical ventilation flow. This effect is demonstrated in Figure 1b when visualizing the aerosol dispersion by blue light illumination when the dummy is wearing a face mask. Consequently, even directly neighboring seats of the dummy show no significant increased aerosol and CO_2_ concentration. All measured aerosol concentrations are diluted to less than 0.9 % with respect to the concentration emitted by the dummy and the CO_2_ concentration is in the range of the background level. The data are summarized in Figure 5. These values are also determined by averaging 5 min. The aerosol background concentration measured in the concert hall was less than 100 p/cm^3^.

**Figure 6:**
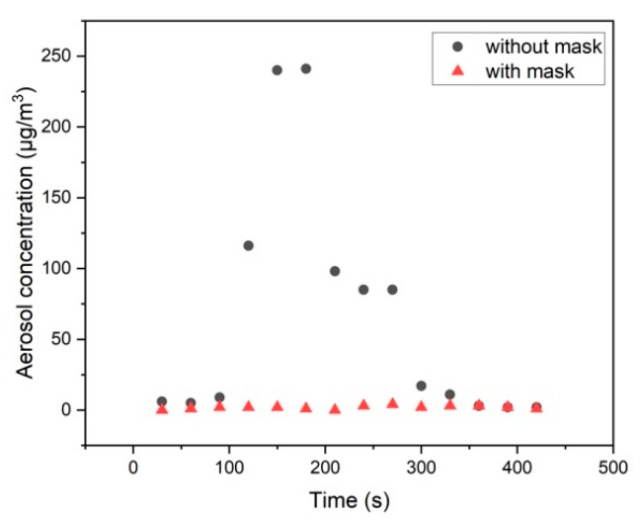
Time-resolved detection of aerosol concentration emitted from the dummy not wearing a mask (black dots) and wearing a surgical mask (red triangles). The distance between dummy and measuring device was d = 0.5 m.

As already discussed above, even slight local horizontal air flow fluctuations may influence the spatial dispersion of aerosol and CO_2_ emitted by the dummy. Therefore, also time-resolved measurements are performed. This is done for the dummy wearing a surgical mask and with unblocked exhalation flow. Typical results of such an experiment are shown in Figure 6. A temporarily varying air stream of $v_{H}<0.01\;m/s$ was measured, the distance between the exhaling dummy and the aerosol and CO_2_ detector was about d = 0.5 m. As can be seen in Figure 6, there is a strong temporal fluctuation of the aerosol concentration obtained—changing between 5 µg/m^3^ which is about the background concentration and up to 240 µg/m^3^—when the dummy is not wearing a mask. However, when the dummy is wearing a face mask then the aerosol concentration is all the time around the background concentration < 5 µg/m^3^. For comparison, a concentration of 25 µg/m^3^ converts to about 500 p/cm^3^ for the DEHS aerosol used here.

In parallel to the aerosol measurement, the CO_2_ concentration was monitored at the same position. In the case of the dummy not wearing a face mask at the beginning of the measurement, the CO_2_ concentration was c = 540 ppm, then increased up to 580 ppm (t = 200 s) and decreased down to 535 ppm for t > 350 s. The CO_2_ concentration values for the measurement while the dummy was wearing a face mask were nearly constant all the time at c = 520 ppm.

**Figure 7:**
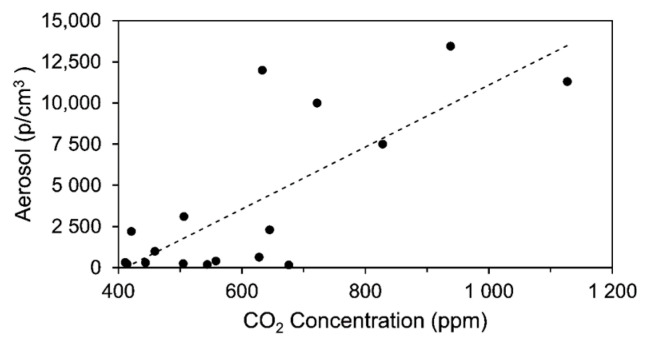
Measured correlation between CO_2_ and aerosol dispersion in the concert hall obtained under real conditions. The Pearson correlation coefficient is r = 0.77.

The simultaneous measurement of aerosol and CO_2_ concentration in the present investigation enables us working out a correlation between CO_2_ and aerosol concentrations under real conditions as they are obtained in the concert hall. The result is shown in Figure 7, indicating a Pearson’s correlation coefficient of r = 0.77 for the given conditions. Under laboratory conditions coefficients up to r = 0.98 have been derived.

## 4. Discussion

Case studies worldwide indicate that airborne aerosols are a major transmission route for indoor SARS-CoV-2 virus infection [16,17,18]. Small airborne particles and aerosols (diameter < 5 µm) can remain up to several hours in the air [43]. So far, most COVID-19 risk assessments for indoor environment are based on calculations applying spreadsheet models including relevant environmental and physiological parameters [8,44,45]. Typical standard settings represent classrooms, office spaces or receptions with space volume < 400 m^3^ and less than 50 persons present. On the other hand, experimental data are available for aerosol emission from a person speaking, singing or shouting [34] that allow developing risk managing strategies and rules, for example for a choir [46].

Our experimental concept presented in this paper enables assessment of infection risk for audience in a concert hall based on experimental data representing the specific situation of the indoor environment under investigation. It simulates a single person present in the audience dispersing aerosol into the air by breathing and analyzing the spatial distribution of the aerosols (indirect infection). It turns out that the most critical factor in the dispersion of aerosols is the installed inhouse ventilation system. Only in case of a vertical fresh air ventilation with a minimum stream velocity of $v_{g,z}=0.05\;m/s$ dispersed aerosols from our dummy are discharged after a short travelling distance (d = 1 m) by the vertical air flow and transported towards the ceiling. If the dummy is surrounded by real persons the vertical ventilation flow will be supported by human body heat but at the same time audience may induce local air flow conditions with a strong temporal fluctuation and flow velocity of $v_{H}=0.01\;m/s$, as obtained in the present investigation. Even such small effects influence the dispersion of emitted aerosols (refer to Figure 4 (seats 22 in row 8 and 9) and Figure 6) which will be very difficult to consider in simulation models frequently used for calculating infection risks. Depending on the initial exhalation velocity even particles with diameter of some micrometers can travel several meters in air before they get captured by the vertical air ventilation stream (refer to Figure 3). Wearing a surgical face mask reduces the horizontal exhalation velocity on very short distance to $v_{H}<0.01\;m/s$ and therefore even particles with diameter up to 40 µm can be captured by the vertical air flow due to their reduced horizontal velocity while larger particles are held back by the mask. Under these conditions even local disturbance of the air flow will not introduce significantly increased aerosol concentrations at neighboring seats (distance to the emitter d = 0.5 m) as can be seen from the data shown in Figure 6. Simultaneous recording of CO_2_ concentration shows a similar pattern.

On the other hand, CO_2_ measurement is a frequently used method for monitoring air quality and ventilation in indoor spaces, respectively. Thus, adding exhaled air to an indoor environment rapidly increases the amount of CO_2_. Concentrations between 400–500 ppm are indicating that the level of ventilation is fairly good, while a concentration of about 800 ppm indicates that 1 % of the air a person is breathing has already been exhaled by another person. This number increases up to 10% for a CO_2_ concentration of about 4400 ppm in the indoor space. Therefore, monitoring exhaled CO_2_ has been discussed as a proxy for estimating COVID-19 infection risk in indoor environments [30,31,33,47,48]. However, there is still no validated proof whether a specific CO_2_ concentration threshold ensures a low COVID-19 infection risk. Using computational fluid dynamics (CFD), Pang et al. [32] simulated a relationship between CO_2_ concentration and infectious aerosols, indicating a higher CO_2_ concentration corresponds to a higher viral load and consequently higher infection risk of COVID-19. This suggests that monitoring CO_2_ could be considered as a simple biomarker of infectious aerosol.

Our experimental results presented in Figure 7 confirm these previously reported suggestions when CO_2_ monitoring is discussed as useful method estimating the quality of ventilation with respect to aerosol dispersion in indoor spaces [31,32,48]. A correlation between the level of CO_2_ and aerosol concentration is obtained when both are emitted simultaneously by the dummy as done in this experimental investigation. An infection risk is directly associated with the fraction of rebreathed air. Therefore, if there are no other CO_2_ sources or sinks and CO_2_ production is only due to human exhalation, spatially resolved monitoring of CO_2_ gives not only information on the efficiency of the installed ventilation system of the indoor environment but also indirect information on dispersion of aerosols. Consequently, under these circumstances CO_2_ monitoring in the audience of a concert hall or theater can work as an indirect marker estimating COVID-19 infection risk. Since this procedure is relatively easy to install, public indoor environments such as concert halls or theaters can use them as an indirect measure for aerosol dispersion with respect to risk assessment and convincing audience that there is efficient ventilation when displaying such data online, e.g., in the foyer after a concert.

## 5. Conclusions

This experimental study demonstrates to our best knowledge for the first time the importance of a vertical fresh air flow ventilation when protecting the audience from viral aerosol dispersion in a concert hall by doing spatially resolved indoor aerosol and CO_2_ measurements. The results indicate that at 1.5 m distance from a potential emitter of aerosols with a diameter of 0.3 µm (our dummy) the aerosol air flow is entrained to the ceiling by the fresh air ventilation system when the vertical air flow is at least $v_{g,z}=0.05\;m/s$. Under such condition, no significant increase of aerosol and CO_2_ concentration is obtained in the concert hall. Longer travelling distances of small and larger particles can be limited by audience wearing a face mask. If the emitting person is wearing a surgical mask and the same fresh air flow conditions apply, for all seats in the direct neighborhood the aerosol and CO_2_ concentration is obtained to be within the air quality category I (CO_2_ < 800 ppm and aerosol < 250 p/cm^3^). Aerosol dispersion in the concert hall does not occur because particles will mostly be directed towards the ceiling by the vertical ventilation flow. Even slight horizontal air flow fluctuations induced by audience will not introduce increased concentrations of aerosol and CO_2_ above the natural background concentration. In addition, the obtained correlation between aerosol and CO_2_ concentration suggests that monitoring the local CO_2_ distribution in the audience can be a very useful technique to estimate the quality and performance of the ventilation system but also as an indirect measure for the risk breathing air that already has been exhaled from another person emitting infectious aerosols.

Furthermore, the results presented in this paper are generally valid for virus transmission by aerosol dispersion. They will be transferable estimating indirect infection risk also to mutations of SARS-CoV-2, for example, B.1.1.7 mutation but also to all other virus types if transmission is dominated by aerosol dispersion.

## Data Availability

The data that support the findings of this study are available from the corresponding author upon reasonable request.
